# A patient with progressive dyspnoea

**DOI:** 10.1007/s12471-015-0718-1

**Published:** 2015-05-28

**Authors:** B.P. Adriaans, I.V. Samarska, B. de Vries, S.C.A.M. Bekkers

**Affiliations:** 1 Department of Cardiology, Maastricht University Medical Center, P. Debyelaan 25, PO Box 5800, 6202 AZ Maastricht, The Netherlands; 2 Department of Pathology, Maastricht University Medical Center, Maastricht, The Netherlands

**Keywords:** Cardiac amyloidosis, Cardiovascular imaging, Cardiovascular magnetic resonance, Echocardiography, Speckle tracking, Pathology

## Abstract

Our case report describes the strong ability of noninvasive diagnostic techniques to detect cardiac involvement in advanced systemic amyloid light chain amyloidosis, which was confirmed at autopsy.

A 63-year-old man was admitted with progressive dyspnoea. Physical examination revealed dullness on percussion and diminished breathing sounds on auscultation of both basal lung fields and pitting oedema of the lower extremities. The electrocardiogram (ECG) showed sinus rhythm, left atrial dilatation, low-voltage QRS complexes in the extremity leads, right QRS axis deviation and clockwise rotation with negative T waves in V5–6 (Fig. 1a). Echocardiography showed mild pericardial but massive pleural effusion, increased biventricular mass, left ventricular ejection fraction (LVEF) 35 % and restrictive diastolic function (E/A > 2, E-wave deceleration time 90 ms) (Fig. 1b, c). Two-dimensional speckle tracking echocardiography showed reduced global longitudinal systolic strain with relative apical sparing (Fig. 1d). Subsequent cardiac magnetic resonance imaging (CMR) showed a typical delayed enhancement pattern with a dark blood pool and globally increased myocardial signal intensity, suggestive of cardiac amyloidosis (Fig. 2a, b). After rectal biopsy, amyloid light chain (AL) amyloidosis was diagnosed. He was treated with diuretics but died 4 months later. At autopsy, primary systemic amyloidosis with extensive cardiac involvement was confirmed. Gross examination showed a marked increase in cardiac mass with atrial dilatation (heart weight 707 g). Microscopy revealed extensive deposition of amyloid with characteristic perimyocytic, interstitial and vascular distribution, confirmed by Congo red staining (Fig. 3a, b). Additionally, a plasma cell dyscrasia was found as associated disease.

**Fig. 1:**
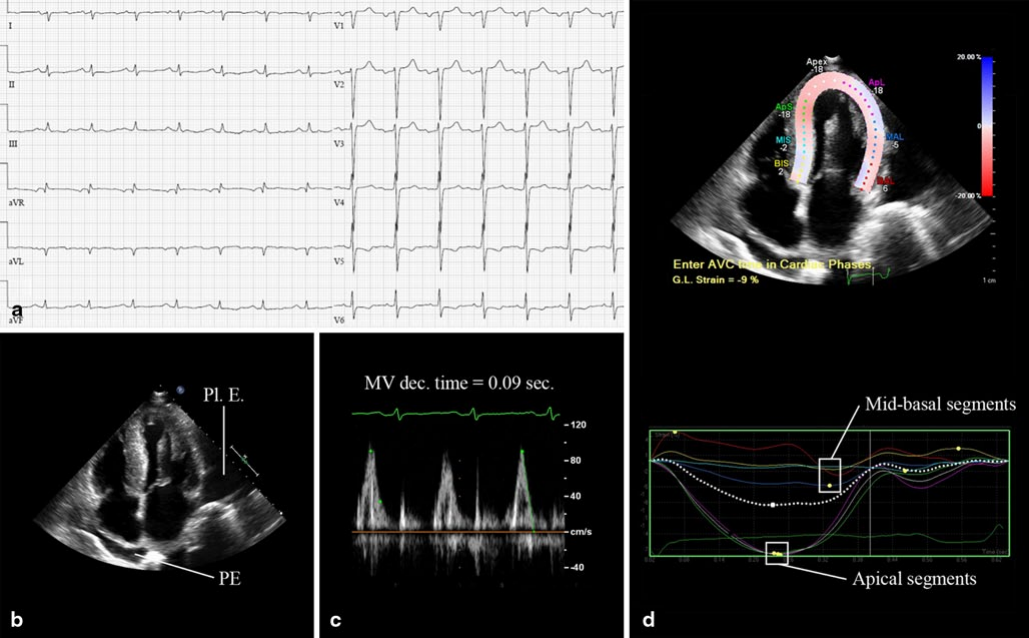
Electrocardiogram (ECG) and echocardiography: **a** ECG showing sinus rhythm, left atrial dilatation, low QRS voltage in the extremity leads, right QRS-axis deviation and clockwise rotation. **b** Two-dimensional echocardiogram: apical four-chamber view showing biventricular hypertrophy, left atrial dilatation, pericardial (*PE*) and pleural effusion (*Pl. E*). **c** Mitral valve pulsed wave Doppler registration, showing restrictive diastolic function. **d** Speckle tracking shows reduced longitudinal strain (− 9 %) of the basal-mid segments with relative apical sparing

**Fig. 2:**
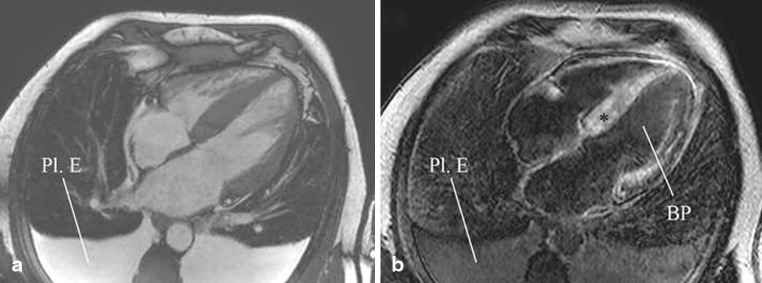
Cardiovascular magnetic resonance imaging: **a** Still frame of a cine horizontal long-axis view, showing ventricular hypertrophy and massive bilateral pleural effusion (*Pl. E*). **b** Delayed enhancement image in horizontal long-axis view obtained 10 min after intravenous gadolinium administration, showing a typical ‘cardiac amyloidosis pattern’: global myocardial enhancement (*) and a dark blood pool (*BP*)

**Fig. 3:**
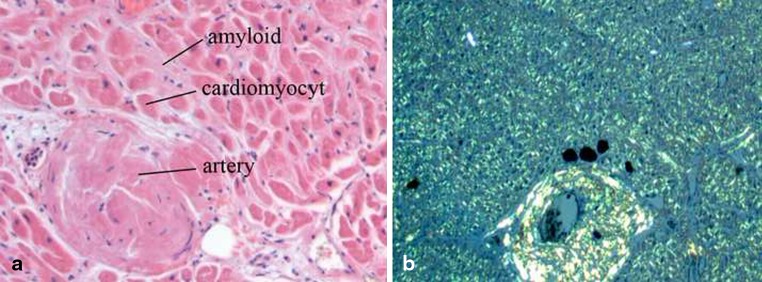
Autopsy findings: **a** Intermediate magnification micrograph at autopsy, hematoxylin eosin staining (HE) staining showing extensive interstitial amyloid deposition between cardiomyocytes as well as vascular amyloid deposition. **b** Congo red staining amyloid shows an *apple-green* birefringence under polarised light

## General discussion

Amyloidosis is characterised by accumulation of insoluble proteins (amyloids) in the extracellular space of different organs. In AL amyloidosis, the most common form, misfolded light chains are produced due to plasma cell dyscrasia. Cardiac amyloid infiltration leads to wall thickening and diastolic dysfunction, resulting in a restrictive cardiomyopathy. The median age at presentation is 60 years. Cardiac involvement carries the worst prognosis, with a median survival of 6 months after the onset of heart failure symptoms [1]. The therapeutic goals are the following: (1) treatment of heart failure and (2) prevention of progressive amyloid deposition. Because beta-blockers and angiotensin-converting enzyme inhibitors are usually not very well tolerated, diuretic therapy is often the sole option to treat heart failure. Dexamethasone combined with melphalan, lenalidomide or bortezomib may increase survival, but rapid disease onset and diagnostic delays contribute to an unfavourable prognosis [2].

## Noninvasive imaging techniques

Endomyocardial biopsy remains the gold standard to diagnose cardiac amyloidosis, but is invasive, associated with complications and limited to experienced centres. Noninvasive cardiac imaging techniques have proven to be of diagnostic value in combination with a high degree of clinical suspicion. The combination of imaging findings of increased ventricular wall thickness and low QRS voltages in the extremity leads on the ECG has a 72 % sensitivity and 91 % specificity to diagnose cardiac amyloidosis [3].

Characteristic echocardiographic findings include atrial enlargement, increased biventricular mass, increased myocardial echogenecity (‘granular speckling pattern’) and diastolic dysfunction with restrictive physiology in the advanced stages of the disease [4]. LVEF may remain normal until late in disease, but longitudinal shortening decreases early. Speckle tracking echocardiography (STE) can measure global and regional longitudinal strain and shows higher apex-to-base strain values with relative apical sparing in cardiac amyloidosis. STE had 93 % sensitivity and 82 % specificity to differentiate cardiac amyloidosis from other hypertrophic myocardial diseases in one study (Fig. 1d) [5].

In cardiac amyloidosis, myocardial gadolinium kinetics after intravenous administration is abnormal due to an expanded extracellular space. Delayed-enhancement CMR commonly shows a global transmural and/or subendocardial enhancement pattern in the advanced stages of the disease [6]. Characteristic for cardiac amyloidosis is that these delayed-enhancement patterns are accompanied with a dark appearance of the blood pool (Fig. 2b). Suboptimal nulling and patchy focal hyper-enhancement may also be observed, but are associated with lesser degrees of amyloid accumulation and a better clinical profile [7]. Delayed-enhancement CMR has a high diagnostic accuracy to diagnose cardiac amyloidosis (sensitivity 88 %, specificity 90 %) and provides prognostic information as well [8]. Visual assessment of T1 using a modified delayed-enhancement CMR pulse sequence is an alternative and simple method to identify abnormal myocardial tissue: myocardial tissue reaches the null crossing (becomes black) at an earlier or same inversion time as the blood pool indicating abnormally low myocardial T1 values and hence abnormally high myocardial gadolinium uptake [9].

Although nuclear imaging is not a first-line investigation, it shows promise to differentiate between cardiac amyloidosis subtypes (cardiac AL and transthyretin-related cardiac amyloidosis (ATTR, mutant and wild types)), which has important therapeutic and prognostic implications [10].

## Pathology

Gross inspection findings at autopsy are generally nonspecific and may mimic other forms of hypertrophic heart disease, such as hypertrophic cardiomyopathy. Atrial enlargement is a constant feature, whereas ventricular dilatation is uncommon. Histologically, amyloid deposits can be highlighted using various stains, of which Congo red is most commonly used for the diagnosis of amyloidosis. Although the origin and compositions of the amyloid deposits may vary, their pathological appearance is identical among forms. With the use of specialised staining techniques, it is possible to differentiate between different forms of amyloidosis, such as in this case where the amyloid depositions were of the AL type.

### Funding

None.

### Conflict of interest

None declared.
